# Supplementary material for: Phase I Trial of Prophylactic Donor-Derived IL-2-Activated NK Cell Infusion after Allogeneic Hematopoietic Stem Cell Transplantation from a Matched Sibling Donor
Source: Cancers (Basel). 2021 May 28;13(11):2673. doi: 10.3390/cancers13112673 (PMC8198961; doi:10.3390/cancers13112673)
Supplement: Supplementary file 1 [file cancers-13-02673-s001.zip › cancers-1214077-supplementary.pdf]

Supplementary table S1: NK cell count and purity at different step of the IL-2 NK manufacturing.

UPN	NK cells (10^6/kg)			NK cell purity (%)			CD3+ cells (10^4/kg) in the final product
	Before isolation	After isolation	After culture in IL-2	Before isolation	After isolation	After culture in IL-2	
#01	12.2	6.7	1.2	6%	92%	90%	1.2
#02	27.2	12.9	2.9	10%	80%	96%	0.3
#03	15.8	11.7	6.4	8%	93%	92%	1.5
#04	12.8	5.7	4.6	4%	81%	97%	0.5
#05	15.4	8.8	4.4	7%	97%	94%	0.3
#06	21.4	10.5	7.6	7%	96%	96%	0.4
#07	16.0	9.5	4.2	8%	94%	96%	0.7
#08	29.5	21.6	4.8	7%	96%	96%	0.6
#09	17.0	12.1	9.5	9%	96%	95%	0.5
#10	4.9	4.1	3.0	5%	97%	92%	0.2
#11	23.2	9.3	4.8	5%	95%	93%	0.0
#12	53.4	31.3	11.3	8%	97%	94%	0.0
#13	38.4	26.4	1.3	13%	94%	95%	0.1
#14	20.1	12.4	5.4	9%	91%	75%	1.2
#15	22.6	10.1	6.4	7%	99%	99%	0.4
#16	17.5	14.8	7.9	10%	97%	98%	0.2

For UPN #01, #02 and #03 only a portion of isolated NK cells have been activated in IL-2 (respectively: 4.6, 6.6,and 4.5 x $10e^6$/kg) because of the low targeted dose. NK cell purity was assessed by gating on viable $CD56^+CD3^-$ cells.

Supplementary table S2: NK cell phenotype before (day+0) vs. after (day+1) IL-2 NK cell infusion

Markers	Frequency of positive NK cell				p
	Day 0		Day 1		
	Median	Range	Median	Range	
DNAM	72.05%	(46.7-96.7)	72.35%	(43.4-86.6)	0.233
NKG2D	93.75%	(55.2-98.7)	93.95%	(52.1-99)	0.894
NKp30	73.9%	(31.3-99.3)	76.75%	(29.8-90.1)	0.470
NKp46	44.95%	(22.6-85.5)	52.9%	(16.3-83.3)	0.910
CD25	0.7%	(0-62.4)	0.6%	(0-63.7)	0.683
CD69	24.1%	(6.3-59.2)	21.3%	(7.8-52.9)	0.966
DR	22.9%	(0.6-85.9)	25.1%	(1-81.8)	0.083
NKp44	5.5%	(1.6-18.3)	6.9%	(2.3-14.7)	0.123
CD57	34.1%	(3.9-53)	38.6%	(21.2-58.9)	0.014
KIR2DL1S1	10.2%	(4.1-15.1)	12.2%	(3-18.5)	0.032
KIR2DL3L2S2	24.8%	(2.5-29.8)	27.5%	(7.7-44.3)	0.014
KIR3DL1	5.1%	(1.4-29.2)	10.2%	(1.7-36.8)	0.014
NKG2C	17.5%	(6.9-27.7)	15.4%	(6.4-38.4)	0.859
NKG2A	68.7%	(50.6-88.6)	66.9%	(45.4-88.4)	0.123
CD107a	15.8%	(9.63-53.1)	27.2%	(12.6-56.4)	0.102
IFN-γ	1.23%	(0.061-23.1)	4.14%	(0.07-8.53)	0.147
MIP-1β	28%	(7.84-69.8)	39.4%	(14.1-68.1)	0.240
TNF-α	2.74%	(0.073-20.3)	6.38%	(0.33-13.5)	0.102
